# Study of Inter- and Intra-varietal Genetic Variability in Grapevine Cultivars

**DOI:** 10.3390/plants11030397

**Published:** 2022-01-31

**Authors:** Alessandra Zombardo, Stefano Meneghetti, Giacomo Morreale, Antonio Calò, Angelo Costacurta, Paolo Storchi

**Affiliations:** 1 Council for Agricultural Research and Economics, Research Centre for Viticulture and Enology, viale Santa Margherita, 80, 52100 Arezzo, Italy; paolo.storchi@crea.gov.it; 2 Accademia Italiana della Vite e del Vino, via Logge degli Uffici Corti 1, 50122 Florence, Italy; stefano.meneghetti72@gmail.com (S.M.); presidenza.aivv.calo@gmail.com (A.C.); accademiaitalianavitevino@gmail.com (A.C.); 3 Council for Agricultural Research and Economics, Research Centre for Viticulture and Enology, viale 28 Aprile, 26, 31015 Conegliano, Italy; giacomo.morreale@crea.gov.it

**Keywords:** Sangiovese, grapevine biodiversity, varietal identification, molecular markers, genetic variability, geographic origin

## Abstract

*Vitis vinifera* includes a large number of cultivars that are further distinguished in biotypes and clones, and it is actually hard to differentiate them, even through complex molecular techniques. In this work, the plant materials of 56 putative Sangiovese and 14 putative Montepulciano biotypes, two of the most widespread black-berried Italian cultivars, were collected in different wine-growing areas of Italy distributed in 13 regions, from north to south. Firstly, the samples were analyzed using SSR markers to have proper varietal identification. According to the results, the genotypes belonged to three different cultivars: Sangiovese, Sanforte, and Montepulciano. Subsequently, the samples were investigated using AFLP, SAMPL, M-AFLP, and I-SSR molecular markers to estimate their intra-varietal genetic variability. The DNA marker-based method used turned out to be performing to bring out the geographic differences among the biotypes screened, and it can therefore be considered as a powerful tool available for all the grapevine varieties.

## 1. Introduction

*Vitis vinifera* L. is one of the oldest known fruit-producing crops that, over the centuries, has undergone strong domestication. This process led to obtaining plants with hermaphroditic flowers (self-fertile), good fruitfulness, propagation capacity, and most of all optimal grape quality for fresh consumption or winemaking [1,2]. The taxonomic reference unit for the grapevine is the variety or cultivar (i.e., a contraction of cultivated variety), and it is estimated that the total number worldwide stands between 6000 and 10,000 [3]. Additionally, a grapevine variety has a higher genetic complexity level: it can be further subdivided into biotypes and clones (namely, officially recognized biotypes). A biotype, or clonal line, is generally selected because standing out for some favorable phenotypic outcomes. These intra-varietal special characteristics can be minor changes in morphological traits, such as bunch compactness and canopy thickness, or macroscopic modifications, such as fruit color (e.g., the grey and white variants of Pinot noir) [4,5,6,7]. It is known that such polymorphisms derive from somatic mutations that occur accidentally after reproduction by vegetative propagation [1,8,9]. Moreover, it has been recently ascertained that intra-varietal differences can arise as plant responses to the environment through epigenetic modifications (clone-dependent DNA methylation patterns) that affect gene expression without altering the DNA sequence [10,11].

In Italy, the first wine-producing country in the world [12], Sangiovese represents the main grapevine variety, with a cultivation area of 54,000 Ha in 2017, covering 8% of the total vineyard area [13] and approximately 7.5 million rooted grafts produced in 2020 [14]. This cultivar is the basic grapevine of the major Tuscan D.O.C. (denomination of controlled origin) and D.O.C.G. (denomination of controlled and guaranteed origin) wines, nationally and internationally renowned (i.e., Chianti Classico, Nobile di Montepulciano, and Brunello di Montalcino). Sangiovese has an ancient, well-attested history in the bibliography of the past, and the first reference to this variety as "Sangioveto" has been recently identified in a treaty about agriculture written by Girolamo Gatteschi (also known as Girolamo da Firenzuola) during a period of imprisonment in Florence in 1552 [15]. Before, Soderini was considered the first author who mentioned Sangiovese as "Sangiogheto" in 1590 [16]. Sangiovese, like many other grapevine cultivars [17,18], is known with different local denominations, according to the area of cultivation (e.g., Brunello in Montalcino, Calabrese in Arezzo, Prugnolo gentile in Montepulciano, Morellino in Grosseto, and Pignolo in Siena, just to mention the cases of some Tuscan wine districts) [19]. In addition, Sangiovese is characterized by high morphological variability, a common feature within the most widely cultivated varieties over the centuries [20,21]. It has a broad population of different biotypes [5,22,23] and the highest number of clones officially registered in the Italian Catalogue of Grapevine Varieties (128, plus three further recorded under the name of Prugnolo gentile) [14]. This ambiguity has often caused varietal classification mistakes [24,25,26,27,28,29]. In the past, Sangiovese was subdivided into two main biotypes: “Sangiovese grosso” (with large berries) and “Sangiovese piccolo” (with small berries) due to a different cluster morphology [28,30]. Subsequently, with the coming of DNA analysis, these two main biotypes showed two distinct genetic profiles: the samples belonging to “Sangiovese grosso” were considered as the true-to-type Sangiovese, while “Sangiovese piccolo” was Sanforte, also known with the synonyms of Maiolica, Sangiovese forte, and Sanvicetro [14,31,32]. It is interesting to mention another peculiar case: Prugnolo gentile, traditionally cultivated in the area of Montepulciano (Siena, Tuscany), was registered in the Italian Catalogue of Grapevine Varieties in 1971 as an independent cultivar, but with the introduction of molecular screenings, it turned out to be indisputably Sangiovese [33]. 

Varietal identification in viticulture has a long tradition. It has always been based mainly on ampelography (from the Greek words *ampelos* and *graphia*, meaning vine description) and ampelometry (the measurement of vine organs) [34,35]. More recent observational methods were introduced later, such as biochemical analyses on either phenolic, terpenic, or protein profiles of the grapes [36,37]. However, these laboratory techniques have some limitations because, as already stated, the vines can often undergo changes in the expression of their phenotypic characteristics [38]. Therefore, a molecular approach is essential since only genetic screening can provide an unequivocal fingerprinting and reveal synonyms or mistaken identifications due to empirical hypotheses. For this purpose, microsatellite markers have high discriminative power and are strongly informative [32,39,40], the reason why a standard set of nine simple sequence repeats (SSR) was developed within the GrapeGen06 European project, and ever since, it has been adopted internationally to discover the identity of a grapevine sample [41]. The use of SSR markers, however, does not allow the detection of intra-varietal differences [6], and thus, other DNA marker-based techniques have been set up to study the genetic variability within *Vitis* species. The most commonly employed are RAPD (random amplified polymorphic DNA), MSAP (methylation-sensitive amplified polymorphism), RT (retrotransposon-based molecular markers), SNP (single-nucleotide polymorphism), IRAP (inter-retrotransposon-amplified polymorphism), AFLP (amplified fragment length polymorphism), M-AFLP (microsatellites amplified fragment length polymorphism), SAMPL (selective amplification of microsatellite polymorphic loci), I-SSR (inter simple sequence repeat), and ISTR (inverse sequence-tagged repeat), and they can also be combined to obtain much more comprehensive information [42,43,44,45,46,47,48,49,50,51,52].

**Figure 1:**
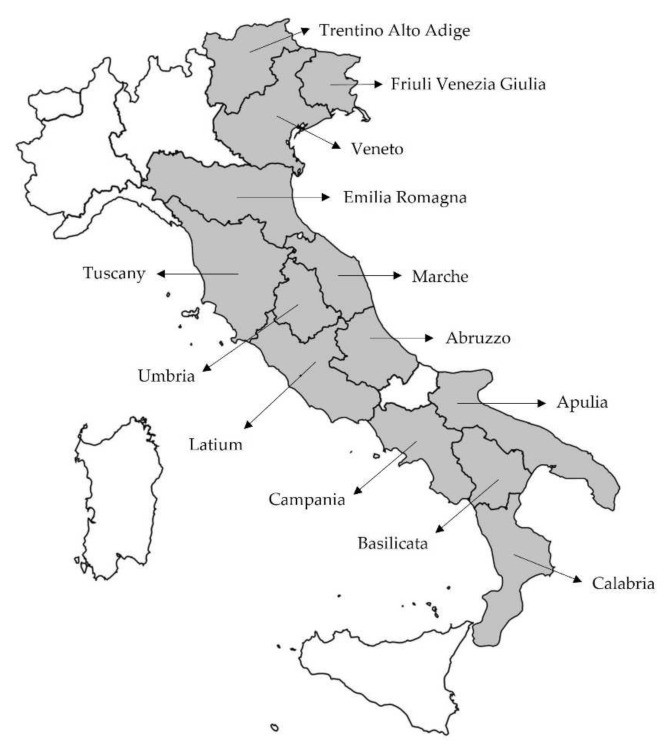
Geographical map of the 13 Italian regions where the putative Sangiovese and Montepulciano samples were collected.

This research aimed at exploring the genetic variability in a broad pool of biotypes allegedly belonging to Sangiovese, the most widespread, black-berried Italian grapevine cultivars [53], and Montepulciano, which was added as the second most cultivated black-berried variety [53] that shares some important wine-growing districts with Sangiovese. In detail, we collected 78 grapevine samples (70, plus seven reference clones and one reference biotype), empirically categorized as putative Sangiovese (56), belonging both to “grosso” and “piccolo” biotypes, and Montepulciano (14) (Table 1). The plant material was recovered in 13 Italian regions, from north to south along much of the peninsula (Figure 1). At first, we verified the true-to-type varietal identity using SSR markers. After establishing that the samples clustered in three different cultivars (i.e., Sangiovese, Sanforte, and Montepulciano), we screened them with four additional molecular markers (i.e., AFLP, SAMPL, M-AFLP, and I-SSR) to estimate the intra-varietal genetic differences. Using a method already proven to be performing in *Vitis* species [54], we wanted to bring out any variations attributable to the geographical origins among the biotypes that may have arisen as an adaptation to the growing environment.

## 2. Results and Discussion

### 2.1. Varietal Identification

**Figure 2:**
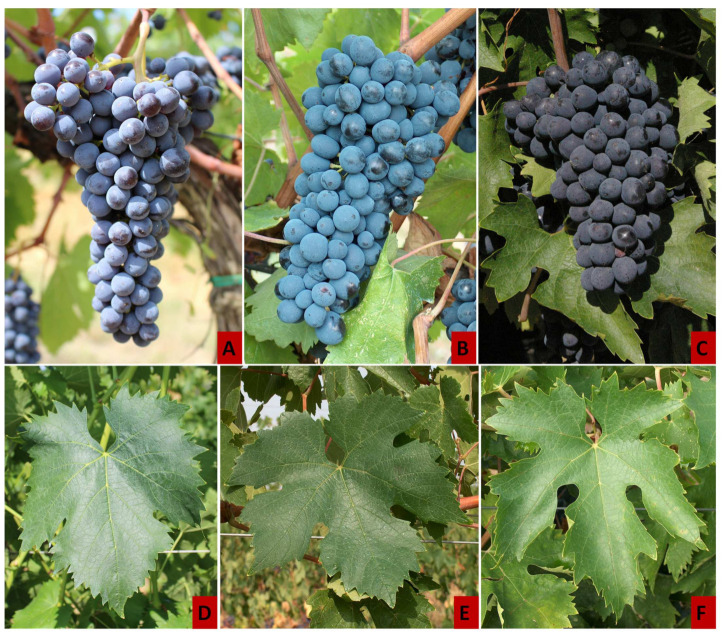
Standard bunch at maturity and standard adult leaf of the three grapevine cultivars revealed by SSR analysis (Sangiovese: (**A**,**D**); Sanforte: (**B**,**E**); Montepulciano: (**C**,**F**)). The photographs are from the grapevine germplasm vineyard collection of CREA—Research Center for Viticulture and Enology in Arezzo (43°47′53′′ N, 11°82′43′′ E, Italy).

According to the results obtained by the SSR marker-assisted screening at 11 microsatellite loci, the 78 samples analyzed can be divided into three variety clusters: 51 Sangiovese (Italian Catalogue of Grapevine Varieties code 218 [14]—*Vitis* International Variety Catalogue code 10680 [31]), 12 Sanforte (Italian Catalogue of Grapevine Varieties code 412—*Vitis* International Variety Catalogue code 7136, registered as Maiolica), and 15 Montepulciano (Italian Catalogue of Grapevine Varieties code 150—*Vitis* International Variety Catalogue code 7949). Figure 2 shows the standard bunch at maturity and the standard adult leaf of the three grapevine cultivars revealed by SSR analysis.

By observing the SSR profiles (reported in Table 2), Sangiovese and Sanforte show identical base pair lengths for both alleles in two SSR loci (VVMD5 and VVMD27), and they share 1 SSR allele in 8 SSR loci (VVS2, VVMD7, VrZAG62, VrZAG79, VMC6E1/ISV2, VMC6F1/ISV3, VMC6G1/ISV4, and VMCNG4b9). The two cultivars may appear genetically similar but the first-degree kinship between them can be excluded since there are no shared alleles in one of the tested loci (VVMD28). Looking further at the values in Table 2, Montepulciano shares one allele in eight SSR loci with Sanforte and only five SSR with at least one common locus with Sangiovese.

Only 51 out of the 63 putative genotypes (45, plus six reference clones) were validated as Sangiovese, while 12 samples (11, plus one reference biotype) turned out to be Sanforte (Table 3). This result can be explained by the fact that, in the past, these two varieties were cultivated together in the vineyards and, sometimes, were even considered two different biotypes of the same cultivar ("Sangiovese grosso" that is “with large berries”, and "Sangiovese piccolo" that is “with small berries”). Moreover, the incorrect names locally assigned by the winegrowers over time helped increase confusion on their real identity. 

The true-to-type correspondence of all the 14 alleged Montepulciano samples was proven by microsatellites markers (Table 3), and the genetic profile of the reference clone RAUSCEDO 7 was confirmed, as expected.

### 2.2. Intra-Varietal Analyses

The samples belonging to Sangiovese, Sanforte, and Montepulciano grapevine varieties (Table 3) were then screened individually (intra-varietal analysis) and together (inter-varietal analysis) using AFLP, SAMPL, M-AFLP, and I-SSR molecular markers. 

A total of 1905 reproducible amplification products were obtained with the four molecular marker systems: 689 AFLPs, 650 SAMPLs, 503 M-AFLPs, and 63 I-SSRs. Of these, 972 (51.2%) were polymorphic: 326 (47.3%) AFLPs, 355 (54.6%) SAMPLs, 268 (53.2%) M-AFLPs, and 23 (36.5%) I-SSRs. The mean numbers of marker loci assayed per single experiment were 62.6, 59.1, 45.7, and 5.7 for AFLP, SAMPL, M-AFLP, and I-SSR markers, respectively.

For all the possible pairwise comparisons of 51 Sangiovese (45 biotypes and 6 clones), 12 Sanforte (12 biotypes), and 15 Montepulciano samples (14 biotypes and 1 clone), a matrix based on Dice’s genetic similarity coefficient (GS) was constructed. The observed total genetic similarity (GS_TOT_) was 0.9056; the genetic similarity estimated within the groups (GS_W_) was 0.9431 for the 51 Sangiovese, 0.9541 for the 12 Sanforte, and 0.9463 for the 15 Montepulciano samples. According to the results, Sanforte was the most homogeneous grapevine variety. The genetic similarity estimated between the groups (GS_B_) was 0.9098 for Sangiovese–Sanforte, 0.8445 for Sangiovese–Montepulciano comparison, and 0.8227 for the Sanforte–Montepulciano comparisons. The data obtained showed that Sangiovese and Sanforte were genetically similar, and it was also found that Montepulciano stands closer to Sangiovese than to Sanforte.

### 2.3. Genetic Diversity

**Figure 3:**
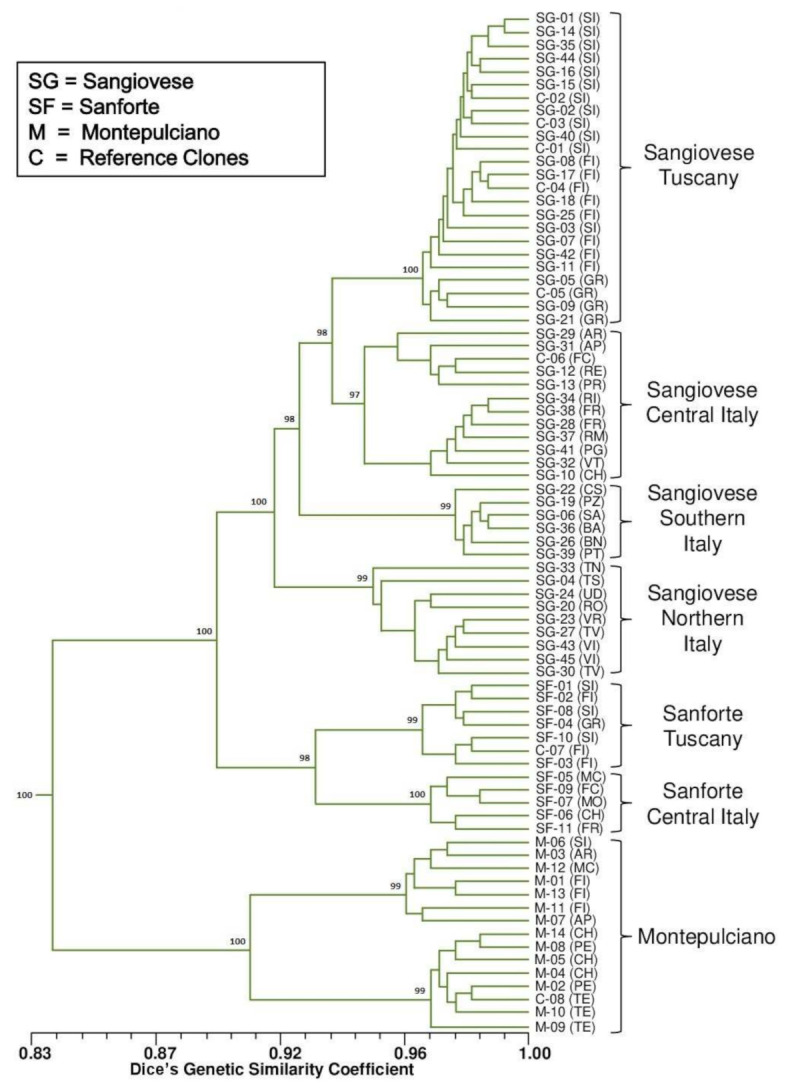
UPGMA dendrogram based on genetic diversity of the 78 samples of Sangiovese, Sanforte, and Montepulciano based on AFLP, SAMPL, M-AFLP, and I-SSR molecular markers. For sample correspondences, see Table 3—Assigned codes). Bootstrap values are indicated. Cophenetic correlation coefficient = 0.9857.

A UPGMA (unweighted pair group method with arithmetic mean) dendrogram based on genetic diversity [57] was obtained using the experimental data (Figure 3). The dendrogram displays the 78 samples grouped into two main clusters: 1) Sangiovese and Sanforte genotypes; 2) Montepulciano genotypes. In any case, the three grapevine varieties are just as clearly separated; these results are consistent with the previous SSR marker-assisted screening (Table 3). Interestingly, several intra-varietal differences related to the area of origin have emerged within each variety cluster.

By observing the lower part of the dendrogram, it can be noted that the 15 samples belonging to the Montepulciano cultivar are grouped according to their region of origin. In particular, there is a sharply defined separation between the samples from Abruzzo, where this grapevine variety is predominant with 56% of the total vineyard area [53] (and also the area of origin of the reference clone RAUSCEDO 7), and Tuscany/Marche, where this cultivar is present but less widespread, with only 19% of vineyard area in Marche and less than 0.5% in Tuscany [53]. 

As intended, the larger cluster is then divided into two sub-clusters: Sangiovese and Sanforte. Sanforte can be discriminated between Tuscan samples and the ones from other regions of Central Italy (Emilia-Romagna, Marche, Abruzzo, Latium). The more complex Sangiovese sub-subcluster brings out pronounced and highly detailed geographical differences. In fact, the 51 samples are split into four main branches: Tuscany, Central Italy, Southern Italy, and Northern Italy. The largest branch is composed of biotypes originating from Tuscany, where this cultivar plays a central role in the regional wine industry and is spread on 64% of the total vineyard area [53]. Within the Tuscan branch, it can be noted that the portion of the province of Grosseto is secluded from the mixed cluster of samples from Siena/Florence. Subsequently, the Central Italy branch (including the regions located in a central position of the Italian peninsula, except for Tuscany) is present and can be partitioned into two main clusters. On one side stands Emilia-Romagna, where Sangiovese is one of the principal varieties linked to Sangiovese di Romagna D.O.C. wine production; on the other side are the samples from other central regions, where Sangiovese is not as strongly widespread (Marche, Umbria, Latium, and Abruzzo). Among the samples from Emilia-Romagna, there is SG-29 (AR), an outlier from Arezzo (a neighboring Tuscan province); the genetic similarity of this outgroup sample with its sub-cluster (SG-31–SG-13) is quite high, more than the average similarity of the whole cluster of Tuscany, a result that is correct both from the statistical and molecular point of view. The Southern Italy branch rightly comprises all the samples from Campania, Basilicata, Calabria, and Apulia. Here too, is inserted an outlier, SG-39 (PT) from Pistoia (Tuscany), which has homogeneity with the samples from the southern regions, where this biotype may have originated. Finally, the Northern Italy branch is divided clearly between the samples from Veneto and those from Trentino Alto Adige/Friuli Venezia Giulia. 

**Figure 4:**
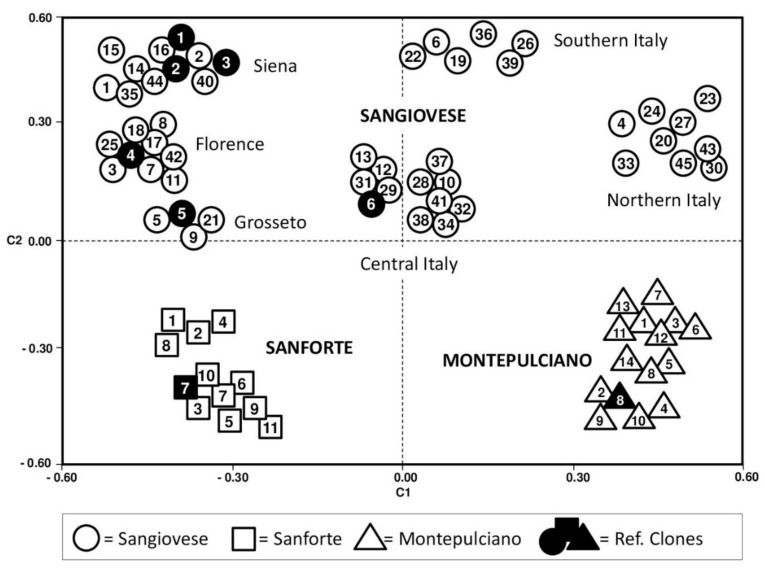
2D centroids of the 78 genotypes screened (Sangiovese—circle, Sanforte—square, and Montepulciano—triangle) based on AFLP, SAMPL, M-AFLP, and I-SSR molecular markers. For sample correspondences, see Table 3—Assigned codes.

The GS matrix estimates and the dendrogram results agree with the principal component analysis (PCA) reported in Figure 4. In the PCA, the first coordinate (C1) captured 46.6% and the second one 37.9% of the variation of the three grapevine genotypes. In particular, Sangiovese samples were well separated from the other cultivars by the coordinate C2, while the coordinate C1 divided Sanforte from Montepulciano, and as for Sangiovese, the samples from Tuscany were distributed according to the geographic origin of the biotypes. As can be seen, the two-dimensional plotting of the centroids showed eight major clusters: (1–3) Sangiovese of Tuscany, in the upper left quadrant, with the samples further split into three provinces (Siena, Florence, and Grosseto); (4) Sangiovese of Central Italy, occupying the center of the graph; (5) Sangiovese of Southern Italy, in the upper right quadrant; (6) Sangiovese of Northern Italy, at the far right of the upper quadrant; (7) Sanforte, in the lower left quadrant; (8) Montepulciano, in the lower right quadrant.

Based on the PCA analysis, the most informative markers in sample discrimination were the primer combination of AFLP *Pst*+AG/*Mse*+CAA with 31 polymorphic markers (51%), the primer combination of SAMPL *As2*/*Mse*+TGG with 32 polymorphic markers (54%), the primer combination M-AFLP I-SSR#02/*M*+AGG with 26 (55%), and the primer combination of I-SSR (TC)_7_ACGG with 4 polymorphic markers (44%) (Table S1).

Collectively considering the results, the combination of different plant-specific molecular markers (i.e., AFLP, SAMPL, M-AFLP, and I-SSR) allowed us to discriminate very efficiently the biotypes belonging to the same variety of *Vitis vinifera*. In particular, the molecular markers used were effective in finding intra-varietal differences since they detect both some repeated regions (different from those of SSR markers) and not-repeated regions adjacent to these repeated regions, thus having a broad view of the grapevine genome. This in-depth genetic characterization has yielded interesting evidence: Sangiovese, Sanforte, and Montepulciano samples were separated quite exhaustively based on their geographical origin (Figure 3 and Figure 4). Genotypes from neighboring areas showed higher genetic similarity compared to the same variety grown in geographically distant regions, highlighting an influence due to environmental stimuli [6,11].

It is well-known that the grapevines, especially several historically more exploited varieties such as Sangiovese [1,58,59], have peculiar phenotypic plasticity (namely, when a genotype can produce different phenotypes). This ability allows the plant to survive and carry forward fruit maturation even in limiting conditions [58,60], thanks to some adaptation strategies that consist of selective modulation of gene expression [59,61]. For this reason, it is possible to hypothesize that the geographic differences found between the biotypes screened have arisen as an adaptation to the pedoclimatic characteristics of the growing area, and probably also in response to any biotic and abiotic stress tackled by the vines [62]. Once a grapevine biotype has adapted to a specific environment via somatic mutation or epigenetic modifications (which occur without causing changes in the DNA sequence), the phenotype can be permanently altered [59]; the transcriptional regulation activity is transmitted through the cell division and, therefore, can be directly inherited from the mother plant [63]. It is important to note that the performance of a specific cultivar or clone in a viticultural district, defined by the genotype–environment interaction, is a distinctive feature of the wine typicality, being one pillar of the "terroir" concept [10,11,64].

Our findings are in agreement with the results of other research successfully conducted with the same DNA marker-based method on other important grapevine varieties, such as Malvasia Nera di Brindisi/Lecce, Malvasia di Candia, Negroamaro, Primitivo/Zinfandel, Grenache Noir/Garnacha Tinta/Cannonau, and Malvasia Istriana [38,49,65], whose effective identification has always been very confused, like that of Sangiovese biotypes.

## 3. Materials and Methods

### 3.1. Plant Materials

Small portions of a woody branch of 56 putative Sangiovese vines (*Vitis vinifera* L. subsp. *vinifera*), belonging both to “Sangiovese grosso” and “Sangiovese piccolo” biotypes, were made available by some farm owners in different wine-growing districts located in 13 different regions along the Italian peninsula (Figure 1). The 56 Sangiovese samples collected are listed in Table 1 with the local denominations assigned and the sampling site (Italian region, provincial acronym). The list includes six official Sangiovese clones (i.e., five from Tuscany (VCR 4, VCR 108, BF 10, MI-BF 50, and VCR 103) and one from Emilia Romagna, VCR 16) [14], and one biotype (Sanvicetro F59 P6 C2, retrieved in Tuscany and preserved in the vineyard-collection of CREA—Research Center for Viticulture and Enology), that were added as references. Similarly, a total of 14 alleged Montepulciano (*Vitis vinifera* L. subsp. *vinifera*) woody branch samples (Table 1) were collected in 3 different regions located in Central Italy (Figure 1; Tuscany, Marche, and Abruzzo). Here too, a reference official clone of Montepulciano (i.e., RAUSCEDO 7, from Abruzzo) was added. Each woody branch was placed in water and sprouted to obtain optimal plant material (fresh young leaves) for subsequent molecular analyses. 

### 3.2. DNA Extraction and Quantification

Genomic DNA was extracted from 4 fresh young leaves, considering three biological replicates per sample. The extraction was performed using the NucleoSpin 8 Plant kit (Macherey-Nagel, Düren, Germany) on the Microlab Starlet Liquid Handling Workstation (Hamilton Robotics, Reno, NV, USA). Genomic DNA was stored undiluted in TE buffer (10 mM Tris-HCl, 1 mM EDTA), pH 8.00 at −20° C. DNA quantification was obtained by FLx800 TBI Fluorometer (BioTek Instruments, Winooski, VT, USA), and PicoGreen dsDNA quantification assay (Invitrogen, Waltham, MA, USA).

### 3.3. Varietal identification

Grapevine samples were genotyped using a set of 11 SSR loci, 6 core loci selected within GenRes 081 European Project (VVS2, VVMD5, VVMD7, VVMD27, VrZAG62 et VrZag79) and other additional 5 (VVMD28, VMC6E1 or ISV2, VMC6F1 or ISV3, VMC6G1 or ISV4, VMCNG4b9), according to internationally accepted standards [41,55,56]. 

The microsatellite PCR reactions were performed on a Microlab Starlet Liquid Handling Workstation (Hamilton Robotics, Reno, NV, USA) using SSR forward (F) 6FAM-labeled primers (VVS2, VVMD7, VVMD27), VIC-labeled primers (VMC6E1, VrZAG62, VVMD5), NED-labeled primers (VMCNG4b9, VrZAG79), PET-labeled primers (VMC6F1, VMC6G1, VVMD28) and SSR reverse (R) unlabeled primers, each at 5 pmol/µL (ThermoFisher Scientific, Waltham, MA, USA), as reported by Meneghetti [65]. 

The microsatellite PCR contained 10 ng genomic DNA; 0.06 μL of VMC6E1, VrZAG79, and VrZAG62 primers (F + R); 0.09 μL of VMCNG4b9, VMC6F1, and VMC6G1 primers (F + R); 0.13 μL of VVS2, VVMD27, and VVMD7 primers (F + R); 0.19 μL of VVMD5 and VVMD28 primers (F + R); 1× PCR buffer (50 mM KCl, 1.5 mM MgCl2, 10 mM Tris–HCl); 4 mM deoxyribonucleotide triphosphates (dNTPs) and 1 U of Taq DNA polymerase (Sigma-Aldrich Corp., St. Louis, MO, USA); and ddH2O to final volume (i.e., 11.5 µL). The PCR was performed in a GeneAmp PCR System 9700 (Applied Biosystems, Italy) with the following conditions: 94 °C for 2 min, 35 cycles at 94 °C for 40 s, 55 °C for 60 s, and 72 °C for 60 s, a final extension at 72 °C for 60 min, and the last step at 4 °C to stop the reaction. SSR polymorphisms were detected on an ABI-3130XL Genetic Analyzer (capillary sequencer, Applied Biosystems, Waltham, MA, USA). The samples were prepared by combining 0.5 μL of labeled PCR, 8 μL Hi-Di™ Formamide, and 0.3 μL of GS500-LIZ size standard (Applied Biosystems, Waltham, MA, USA). The software used for the analyses was GeneMapper v.4.1 (Applied Biosystems, Waltham, MA, USA) with a *Vitis vinifera* microsatellite bin set of 11 standard loci.

After having established the correct genetic identity, each of the 78 samples was renamed with an assigned code (Table 3) compound with a letter indicating the variety, a progressive number (SG-01/45 for Sangiovese; SF-01/11 for Sanforte; M-01/14 for Montepulciano; C-01/07 for reference clones or reference biotype) and the provincial acronym as the geographic origin (in brackets), in view of subsequent data processing.

### 3.4. Intra-Varietal Analyses

The genetic variability within the same grapevine cultivar was investigated by AFLP (amplified fragment length polymorphism), SAMPL (selective amplification of microsatellite polymorphic loci), M-AFLP (microsatellites amplified fragment length polymorphism), and I-SSR (inter simple sequence repeat) molecular markers [54,66].

The restriction of DNA was performed by EcoRI, PstI, and MseI enzymes with EcoRI, PstI, and MseI adapters (a and b, see Table S1); T4 ligase was used as a ligation enzyme (all the reagents were obtained from New England Biolabs, Ipswich, MA, USA). Pre-amplification was performed using 5 µL of seven-fold diluted, digested, and ligated DNAs in 20 µL of reaction mixture containing 75 ng of EcoRI+1 (or PstI+1) and MseI+1 primers (one selective nucleotide), 1x PCR buffer (50 mM KCl, 1.5 mM MgCl2, 10 mM Tris–HCl), 10 mM dNTPs, and 1 U of Taq DNA polymerase (Sigma-Aldrich Corp., St. Louis, MO, USA). The AFLP-based and I-SSR analyses were performed using a 6FAM/VIC/NED/PET labeled EcoRI+3 (or PstI+2) primer and an unlabeled MseI+3 primer (three selective nucleotides). Primers and primer combinations are reported in Table S1.

A binary presence or absence (1 vs. 0) matrix was created for AFLP, SAMPL, M-AFLP, and I-SSR markers and for each genotype. Molecular markers were defined by a standard ladder using the GeneMapper software with some reference DNA genotypes and automatically visualized using the software of ABI-3130XL capillary sequencer. 

### 3.5. Genetic Similarity

Genetic similarity (GS_TOT_) estimates among individuals were calculated in all possible pairwise comparisons using Dice’s genetic coefficient by NTSYS software and UPGMA algorithm [38,49]. GS was calculated within (GS_W_) and between (GS_B_) sample clusters and marker systems (AFLP, SAMPL, M-AFLP, and I-SSR). The cluster analysis of GS (dendrograms, PCA centroids) was performed according to the UPGMA algorithm using the NTSYS software [57]. The molecular profiles of the Sangiovese and Montepulciano clones and the Sanforte biotype (SSR, AFLP, I-SSR, M-AFLP, and SAMPL) were used as a reference only.

## 4. Conclusions

The system based on the four AFLP, SAMPL, M-AFLP, and I-SSR molecular markers used turned out to be performing to bring out the geographic differences among the biotypes comprised in this research work. This method can be considered a powerful tool available for all *Vitis vinifera* cultivars, especially those characterized by high morphological variability. It finds applications where SSR analysis is not sensitive enough, such as biotype intra-varietal screening or if more complex “omics” approaches cannot be used. 

## Figures and Tables

**Table 1:** List of the 78 grapevine samples screened: 56 putative Sangiovese, 14 putative Montepulciano, 7 reference clones, and 1 reference biotype are highlighted in bold red.

Sample No.	Sample Name	Geographic Origin Region (Province)	No.	Sample Name	Geographic Origin Region (Province)
1	Sangiovese Chianti 1	Tuscany (AR)	40	Sangiovese Emiliano	Emilia Romagna (RE)
2	Morellino di Toscana	Tuscany (FI)	41	Sangiovese Marchigiano	Marche (AP)
3	Morellino Precoce	Tuscany (FI)	42	Sanvicetro Marche	Marche (MC)
4	Sangiovese Brunello 1	Tuscany (FI)	43	Sangiovese Perugino	Umbria (PG)
5	Sangiovese Chianti 2	Tuscany (FI)	44	Brunello Abruzzese	Abruzzo (CH)
6	Sangiovese Chianti 3	Tuscany (FI)	45	Sanvicetro Abruzzese	Abruzzo (CH)
7	Sangiovese Classico	Tuscany (FI)	46	Brunellino Rosso	Latium (FR)
8	Sangiovese Toscano 1	Tuscany (FI)	47	Morellino Romano	Latium (FR)
9	Sanvicetro Fiorentino	Tuscany (FI)	48	Sanvicetro Laziale 1	Latium (FR)
10	Sanvicetro Morellino	Tuscany (FI)	49	Brunello Montalcino 2	Latium (RI)
11	Morellino di Scansano	Tuscany (GR)	50	Brunello di Frascati	Latium (RM)
12	Sangiovese Rosso	Tuscany (GR)	51	Sanvicetro Laziale 2	Latium (VT)
13	Sangiovese Scansano 1	Tuscany (GR)	52	Sangiovese Campano	Campania (BN)
14	Sangiovese Scansano 2	Tuscany (GR)	53	Sangiovese Brunello 2	Campania (SA)
15	Sangiovese Prugnolo	Tuscany (PT)	54	Morellino di Potenza	Basilicata (PZ)
16	Brunello di Montalcino	Tuscany (SI)	55	Sangiovese Calabro	Calabria (CS)
17	Brunello Montalcino 1	Tuscany (SI)	56	Sangiovese Nostrano	Apulia (BA)
18	Montalcino Prugnolo	Tuscany (SI)	57	**Sangiovese VCR 4**	Tuscany (FI)
19	Sangiovese Gaiole	Tuscany (SI)	58	**Sangiovese VCR 108**	Tuscany (GR)
20	Sangiovese Grosso	Tuscany (SI)	59	**Sangiovese BF 10**	Tuscany (SI)
21	Sangiovese Lungo	Tuscany (SI)	60	**Sangiovese MI-BF 50**	Tuscany (SI)
22	Sangiovese Montepulciano	Tuscany (SI)	61	**Sangiovese VCR 103**	Tuscany (SI)
23	Sangiovese Morellino	Tuscany (SI)	62	**Sangiovese VCR 16**	Emilia Romagna (FC)
24	Sangiovese Senese	Tuscany (SI)	63	**Sanvicetro F59 P6 C2**	Tuscany (FI)
25	Sangiovese Toscano 2	Tuscany (SI)	64	Montepulciano di Toscana	Tuscany (AR)
26	Sanvicetro di Toscana	Tuscany (SI)	65	Montepulciano	Tuscany (FI)
27	Sanvicetro Toscano	Tuscany (SI)	66	Montepulciano Grosso	Tuscany (FI)
28	Sangiovese Rovigo	Veneto (RO)	67	Montepulciano Sangiovese	Tuscany (FI)
29	Sangiovese Susegana	Veneto (TV)	68	Montepulciano Toscano	Tuscany (SI)
30	Sangiovese Trevigiano	Veneto (TV)	69	Montepulciano Marche 1	Marche (AP)
31	Morellino Berico	Veneto (VI)	70	Montepulciano Marche 2	Marche (MC)
32	Sanvicetro Piccolo	Veneto (VI)	71	Montepulciano Abruzzese	Abruzzo (CH)
33	Morellino Gentile	Veneto (VR)	72	Montepulciano Classico 1	Abruzzo (CH)
34	Sangiovese Trentino	Trentino Alto Adige (TN)	73	Montepulciano Rotondo	Abruzzo (CH)
35	Sangiovese Carsico	Friuli Venezia Giulia (TS)	74	Montepulciano Classico 2	Abruzzo (PE)
36	Sangiovese Friulano	Friuli Venezia Giulia (UD)	75	Montepulciano Glabro	Abruzzo (PE)
37	Sanvicetro Emiliano	Emilia Romagna (FC)	76	Montepulciano Nobile	Abruzzo (TE)
38	Sangiovese Giove	Emilia Romagna (MO)	77	Montepulciano Teramano	Abruzzo (TE)
39	Sangiovese Montalcino	Emilia Romagna (PR)	78	**Montepulciano Rauscedo 7**	Abruzzo (TE)

Provincial acronyms listed by region: Tuscany (AR—Arezzo, FI—Firenze, GR—Grosseto, PT—Pistoia, and SI—Siena); Trentino Alto Adige (TN—Trento); Friuli Venezia Giulia (TS—Trieste and UD—Udine); Veneto (RO—Rovigo, VI—Vicenza, VR—Verona, and TV—Treviso); Emilia-Romagna (FC—Forlì-Cesena, PR—Parma, MO—Modena, and RE—Reggio Emilia); Umbria (PG—Perugia); Marche (AP—Ascoli Piceno and MC—Macerata); Abruzzo (CH—Chieti, PE—Pescara, and TE—Teramo); Latium (FR—Frosinone, RI—Rieti, RM—Roma, and VT—Viterbo); Campania (BN—Benevento and SA—Salerno); Basilicata (PZ—Potenza); Apulia (BA—Bari); Calabria (CS—Cosenza).

**Table 2:** Allele sizes at 11 SSR loci of Sangiovese, Sanforte, and Montepulciano (lengths are expressed in base pairs). The varietal identification was carried out according to [41,55,56].

SSR locus	Sangiovese		Sanforte		Montepulciano	
VVS2	133	133	133	151	133	145
VVMD5	226	236	226	236	226	228
VVMD7	239	263	239	249	249	249
VVMD27	179	185	179	185	189	194
VVMD28	237	247	239	239	237	247
VrZAG62	193	195	195	201	189	199
VrZAG79	242	258	250	258	250	250
VMC6E1/ISV2	143	165	141	143	141	169
VMC6F1/ISV3	139	139	131	139	139	145
VMC6G1/ISV4	177	197	177	187	187	191
VMCNG4b9	158	168	150	168	168	176

**Table 3:** Varietal identification by SSR markers of the 78 samples analyzed and geographic cluster set using AFLP, SAMPL, M-AFLP, and I-SSR molecular markers. The assigned code is composed of the initial letters of the variety progressive number (origin: provincial acronym). The 7 reference clones and 1 reference biotype are highlighted in bold red.

Sample Name (Sample No.)	Genotype ID by SSR	Assigned Code (Origin)	Geographic Cluster
**Sangiovese BF 10 (59)**	**Sangiovese**	**C-01 (SI)**	**Tuscany**
**Sangiovese MI-BF 50 (60)**	**Sangiovese**	**C-02 (SI)**	**Tuscany**
**Sangiovese VCR 103 (61)**	**Sangiovese**	**C-03 (SI)**	**Tuscany**
**Sangiovese VCR 4 (57)**	**Sangiovese**	**C-04 (FI)**	**Tuscany**
**Sangiovese VCR 108 (58)**	**Sangiovese**	**C-05 (GR)**	**Tuscany**
Sangiovese Montepulciano (22)	Sangiovese	SG-01(SI)	Tuscany
Sangiovese Gaiole (19)	Sangiovese	SG-02 (SI)	Tuscany
Sangiovese Lungo (21)	Sangiovese	SG-03 (SI)	Tuscany
Sangiovese Scansano 1 (13)	Sangiovese	SG-05(GR)	Tuscany
Morellino di Toscana (2)	Sangiovese	SG-07 (FI)	Tuscany
Sanvicetro Morellino (10)	Sangiovese	SG-08 (FI)	Tuscany
Sangiovese Scansano 2 (14)	Sangiovese	SG-09 (GR)	Tuscany
Sangiovese Classico (7)	Sangiovese	SG-11 (FI)	Tuscany
Montalcino Prugnolo (18)	Sangiovese	SG-14 (SI)	Tuscany
Brunello di Montalcino (16)	Sangiovese	SG-15 (SI)	Tuscany
Sangiovese Toscano 2 (25)	Sangiovese	SG-16 (SI)	Tuscany
Sangiovese Chianti 2 (5)	Sangiovese	SG-17 (FI)	Tuscany
Sangiovese Chianti 3 (6)	Sangiovese	SG-18 (FI)	Tuscany
Morellino di Scansano (11)	Sangiovese	SG-21 (GR)	Tuscany
Morellino Precoce (3)	Sangiovese	SG-25 (FI)	Tuscany
Sangiovese Chianti 1 (1)	Sangiovese	SG-29 (AR)	Tuscany
Sanvicetro Toscano (27)	Sangiovese	SG-35 (SI)	Tuscany
Sangiovese Prugnolo (15)	Sangiovese	SG-39 (PT)	Tuscany
Brunello Montalcino 1 (17)	Sangiovese	SG-40 (SI)	Tuscany
Sangiovese Brunello 1 (4)	Sangiovese	SG-42 (FI)	Tuscany
Sangiovese Grosso (20)	Sangiovese	SG-44 (SI)	Tuscany
Sangiovese Carsico (35)	Sangiovese	SG-04 (TS)	Northern Italy
Sangiovese Rovigo (28)	Sangiovese	SG-20 (RO)	Northern Italy
Morellino Gentile (33)	Sangiovese	SG-23 (VR)	Northern Italy
Sangiovese Friulano (36)	Sangiovese	SG-24 (UD)	Northern Italy
Sangiovese Trevigiano (30)	Sangiovese	SG-27 (TV)	Northern Italy
Sangiovese Susegana (29)	Sangiovese	SG-30 (TV)	Northern Italy
Sangiovese Trentino (34)	Sangiovese	SG-33 (TN)	Northern Italy
Sanvicetro Piccolo (32)	Sangiovese	SG-43 (VI)	Northern Italy
Morellino Berico (31)	Sangiovese	SG-45 (VI)	Northern Italy
**Sangiovese VCR 16 (62)**	**Sangiovese**	**C-06 (FC)**	**Central Italy**
Sangiovese Emiliano (40)	Sangiovese	SG-12 (RE)	Central Italy
Sangiovese Montalcino (39)	Sangiovese	SG-13 (PR)	Central Italy
Brunellino Rosso (46)	Sangiovese	SG-28 (FR)	Central Italy
Sanvicetro Laziale 2 (51)	Sangiovese	SG-32 (VT)	Central Italy
Brunello Montalcino 2 (49)	Sangiovese	SG-34 (RI)	Central Italy
Brunello di Frascati (50)	Sangiovese	SG-37 (RM)	Central Italy
Morellino Romano (47)	Sangiovese	SG-38 (FR)	Central Italy
Brunello Abruzzese (44)	Sangiovese	SG-10 (CH)	Central Italy
Sangiovese Marchigiano (41)	Sangiovese	SG-31 (AP)	Central Italy
Sangiovese Perugino (43)	Sangiovese	SG-41 (PG)	Central Italy
Sangiovese Brunello 2 (53)	Sangiovese	SG-06 (SA)	Southern Italy
Morellino di Potenza (54)	Sangiovese	SG-19 (PZ)	Southern Italy
Sangiovese Calabro (55)	Sangiovese	SG-22 (CS)	Southern Italy
Sangiovese Campano (52)	Sangiovese	SG-26 (BN)	Southern Italy
Sangiovese Nostrano (56)	Sangiovese	SG-36 (BA)	Southern Italy
**Sanvicetro F59 P6 C2 (63)**	**Sanforte**	**C-07 (FI)**	**Tuscany**
Sanvicetro di Toscana (26)	Sanforte	SF-01 (SI)	Tuscany
Sangiovese Toscano 1 (8)	Sanforte	SF-02 (FI)	Tuscany
Sanvicetro Fiorentino (9)	Sanforte	SF-03 (FI)	Tuscany
Sangiovese Rosso (12)	Sanforte	SF-04 (GR)	Tuscany
Sangiovese Morellino (23)	Sanforte	SF-08 (SI)	Tuscany
Sangiovese Senese (24)	Sanforte	SF-10 (SI)	Tuscany
Sanvicetro Abruzzese (45)	Sanforte	SF-06 (CH)	Central Italy
Sanvicetro Marche (42)	Sanforte	SF-05 (MC)	Central Italy
Sangiovese Giove (38)	Sanforte	SF-07 (MO)	Central Italy
Sanvicetro Emiliano (37)	Sanforte	SF-09 (FC)	Central Italy
Sanvicetro Laziale 1 (48)	Sanforte	SF-11 (FR)	Central Italy
**Montepulciano Rauscedo 7 (78)**	**Montepulciano**	**C-08 (TE)**	**Abruzzo**
Montepulciano Glabro (75)	Montepulciano	M-02 (PE)	Abruzzo
Montepulciano Classico 1 (72)	Montepulciano	M-04 (CH)	Abruzzo
Montepulciano Abruzzese (71)	Montepulciano	M-05 (CH)	Abruzzo
Montepulciano Classico 2 (74)	Montepulciano	M-08 (PE)	Abruzzo
Montepulciano Teramano (77)	Montepulciano	M-09 (TE)	Abruzzo
Montepulciano Nobile (76)	Montepulciano	M-10 (TE)	Abruzzo
Montepulciano Rotondo (73)	Montepulciano	M-14 (CH)	Abruzzo
Montepulciano Marche 1 (69)	Montepulciano	M-07 (AP)	Marche
Montepulciano Marche 2 (70)	Montepulciano	M-12 (MC)	Marche
Montepulciano Grosso (66)	Montepulciano	M-01 (FI)	Tuscany
Montepulciano di Toscana (64)	Montepulciano	M-03 (AR)	Tuscany
Montepulciano Toscano (68)	Montepulciano	M-06 (SI)	Tuscany
Montepulciano (65)	Montepulciano	M-11 (FI)	Tuscany
Montepulciano Sangiovese (67)	Montepulciano	M-13 (FI)	Tuscany

SG—Sangiovese; SF—Sanforte; M—Montepulciano; C (control)—reference clones and biotypes.

## Data Availability

The original data presented in this research work are stored in the databases of CREA—Research Centre for Viticulture and Enology.

## Supplementary Materials

The following supporting information can be downloaded at https://www.mdpi.com/article/10.3390/plants11030397/s1, Table S1: Primers and primer combinations: AFLP, SAMPL, and M-AFLP primer combinations and I-SSR primers used to study the intra-varietal genetic variability of Sangiovese, Sanforte, and Montepulciano.
