# Supplementary material for: Study of Inter- and Intra-varietal Genetic Variability in Grapevine Cultivars
Source: Plants (Basel). 2022 Jan 31;11(3):397. doi: 10.3390/plants11030397 (PMC8839970; doi:10.3390/plants11030397)
Supplement: Supplementary file 1 [file plants-11-00397-s001.zip › plants-1544497-supplementary.pdf]

Table S1. Primers and primer combinations: AFLP, SAMPL and M-AFLP primer combinations and ISSR primers used to study the intra-varietal genetic variability of Sangiovese, Sanforte, and Montepulciano. E, P and M are the Eco-RI, Pst-I and Mse-I AFLP primers respectively, with the two or three selective nucleotides (A, G, C and T), As1 and As2 are the SAMPL primers, while M-AFLPs are the SSRs/ISSRs and AFLP (P or E or M) primer combinations.

No.	AFLP	SAMPL	M-AFLP	ISSR
1	*P*+AC/*M*+AAG	*As*1/*M*+AGC	*E*+CGT/ISSR#06	$(CA)_7$GATC
2	*P*+AG/*M*+CGG	*As*1/*M*+AGT	*E*+AAT/ISSR#08	$(CA)_7$ATCT
3	*P*+AT/*M*+CAA	*As*1/*M*+AGG	*E*+CAA/ISSR#13	$(CA)_7$AGTC
4	*P*+AA/*M*+CGA	*As*1/*M*+ACC	ISSR#02/*M*+AGG	$(TC)_7$ACAT
5	*P*+AG/*M*+CAA	*As*1/*M*+CTC	*E*+TGA/ISSR#19	$(TC)_7$AGTC
6	*E*+CTT/*M*+ATG	*As*1/*M*+CGT	*P*+AC/ISSR#05	$(TC)_7$ACGG
7	*E*+ATG/*M*+CAG	*As*2/*M*+AGA	*P*+AT/ISSR#22	GGTC$(AC)_7$
8	*E*+CGT/*M*+CTG	*As*2/*M*+TGG	VVMD7f/*M*+ATC	CGTC$(AC)_7$
9	*E*+CAT/*M*+CCT	*As*2/*M*+CAG	VVS2f/*M*+CGT	AGAT$(TC)_7$
10	*E*+AGA/*M*+ATA	*As*2/*M*+CGA	ISSR#07/*M*+ACG	GTGC$(TC)_7$
11	*E*+TCA/*M*+CAG	*As*2/*M*+CGT	ISSR#11/*M*+ACC	CAGC$(TA)_7$
